# Comparing Isocitrate Dehydrogenase Inhibitors with Procarbazine, Lomustine, and Vincristine Chemotherapy for Oligodendrogliomas

**DOI:** 10.3390/cancers17233880

**Published:** 2025-12-04

**Authors:** Gerardo Duran, Diego Pichardo-Rojas, Ahmed Hashim Ali, Peter Passias, Angela Downes, Wilson Z. Ray, Gregory J. Zipfel, Hakeem J. Shakir, Andrew Bauer, Andrew Jea, Ian F. Dunn, Jeffrey A. Zuccato, Christopher S. Graffeo, M. Burhan Janjua

**Affiliations:** 1 Department of Neurosurgery, University of Oklahoma College of Medicine, Oklahoma City, OK 73104, USA; gerardo-duran@ou.edu (G.D.); aha13009@creighton.edu (A.H.A.); ian-dunn@ou.edu (I.F.D.); jeffrey-zuccato@ou.edu (J.A.Z.); graffeo@gmail.com (C.S.G.); 2 Instituto Nacional de Neurología y Neurocirugía, Mexico City 14269, Mexico; diego.pichardo@uabc.edu.mx; 3 Department of Neurosurgery, Texas Health Physicians Group, Arlington, TX 76014, USA; 4 Department of Neurosurgery and Orthopedic Surgery, Duke University School of Medicine, Durham, NC 27710, USA; 5 Department of Neurosurgery, Washington University School of Medicine, St. Louis, MO 63110, USA

**Keywords:** oligodendroglioma, isocitrate dehydrogenase inhibitor, procarbazine lomustine vincristine chemotherapy, targeted therapy

## Abstract

The abstract has been submitted for presentation to the AANS 2026 meeting being held in San Antonio, TX, USA. **Introduction:** Oligodendrogliomas are an uncommon subset of gliomas that are molecularly defined by 1p/19q codeletion in the setting of an isocitrate dehydrogenase (IDH) 1/2 mutation. Standard-of-care management involves maximal safe resection followed by adjuvant chemoradiation with procarbazine, lomustine, and vincristine (PCV). Although PCV confers a durable survival advantage, treatment-limiting toxicity is common and often necessitates discontinuation. IDH inhibitors such as vorasidenib have demonstrated promising efficacy and more favorable tolerability profiles, but a paucity of comparative data across therapeutic classes limits optimal treatment decision-making. **Methods:** A systematic search was conducted through to 7 March 2025 in accordance with the Preferred Reporting Items for Systematic Reviews and Meta-Analyses extension for Scoping Reviews (PRISMA-ScR). Eligible studies included adult patients (≥18 years) with IDH-mutant, 1p/19q-codeleted oligodendrogliomas treated with PCV chemotherapy or IDH inhibitors and with a minimum follow-up of 12 months. Outcomes of interest included overall survival (OS), progression-free survival (PFS), and grade ≥ 3 adverse events (AEs) that led to treatment discontinuation. **Results:** Twenty-eight studies met the inclusion criteria, with a total of 406 patients. All 406 patients carried a confirmed diagnosis of oligodendroglioma. For mixed-histology cohorts, only oligodendroglioma-specific data were extracted and analyzed. Among PCV cohorts, median PFS ranged from 24.3 months to 8.4 years and median OS was reported up to 14.7 years in long-term follow-up from RTOG 9402 and EORTC 26951. Grade ≥ 3 AEs resulted in treatment discontinuation in 65–70% of patients, primarily due to hematologic or neurologic events. In comparison, vorasidenib achieved a median PFS of 27.7 months in the phase III INDIGO trial (HR 0.39; 95% CI 0.27–0.56; *p* < 0.001), with median OS not yet reached at 14.2 months of follow-up. Grade ≥ 3 AEs occurred in 22.8% of patients and led to treatment discontinuation in only 1–3%, primarily due to asymptomatic transaminitis. Early real-world data from expanded-access programs similarly support these tolerability findings. **Conclusions:** While PCV chemotherapy remains the standard-of-care systemic therapy for oligodendroglioma supported by mature survival data, IDH inhibitors represent a mechanistically targeted alternative with encouraging early-phase outcomes and a significantly improved safety profile. Direct comparison across these regimens is constrained by differences in study design and limited long-term OS data for IDH inhibitors. Prospective head-to-head trials are essential for defining the optimal therapeutic sequence in this evolving treatment landscape. In the interim, we provide a recommend approach for current use.

## Simple Summary

Oligodendroglioma is an uncommon brain tumor that often affects younger and middle-aged adults and can progress slowly over many years. The current treatment approach relies on surgery followed by radiation and a three-drug chemotherapy regimen known to improve long-term survival, although many patients struggle with significant treatment-related side effects. Recently, new medicines have been developed that directly target the genetic changes that drive these tumors. These targeted treatments are generally easier to tolerate and may postpone the need for radiation or chemotherapy, but they have not yet been compared directly with the long-established regimen. In this review, we gathered and evaluated all available studies on both approaches to clarify what is known about their benefits, their risks, and the strength of evidence supporting each option. We found that the traditional regimen remains the only therapy with proven survival advantages measured over many years, while the targeted medicines offer early signs of disease control with fewer severe side effects. These findings highlight the need for future studies that directly compare the two strategies. They also provide patients and clinicians with a clearer foundation for thoughtful, individualized treatment decisions.

## 1. Introduction

Oligodendrogliomas account for approximately 5–15% of all gliomas and 3–4% of all brain tumors [1]. The majority are classified as WHO grade 2, while the more aggressive grade 3 tumors comprise about 20% of cases. Historically they were defined by histopathological features, but the 2021 WHO diagnostic criteria defined oligodendroglioma by molecular markers, specifically the co-occurrence of an IDH1 or IDH2 mutation and a 1p/19q codeletion [2]. Under the 2021 WHO classification, oligodendroglioma is defined exclusively by the presence of an IDH1 or IDH2 mutation together with whole-arm 1p/19q codeletion [3]. This framework marks the transition from morphology-based diagnosis to an integrated molecular system, improving diagnostic reproducibility and aligning clinical decision-making with underlying tumor biology [4]. Oligodendrogliomas account for approximately five to fifteen percent of all primary gliomas and show the highest incidence among adults in their fourth and fifth decades of life [1]. Population-based surveillance studies report a modest male predominance and increased incidence in individuals of Northern European ancestry [3,4,5]. Their natural history is relatively indolent, with median survival frequently exceeding ten to fifteen years in modern cohorts, even in cases with subtotal resection [3,5]. These epidemiologic features underscore the importance of treatment strategies that balance long-term disease control with cumulative toxicity.

In contemporary molecularly defined cohorts, most oligodendrogliomas are classified as WHO grade 2, whereas grade 3 tumors comprise approximately twenty percent of cases [1,3]. Population-based analyses demonstrate that grade retains prognostic significance despite uniform IDH mutation and 1p/19q codeletion status [2,5]. Histopathologic assessment remains essential, as grade 3 tumors typically exhibit higher mitotic activity, increased Ki-67 proliferation indices, microvascular proliferation, and architectural heterogeneity, features associated with earlier progression and a greater likelihood of requiring multimodal therapy [2,5]. In contrast, grade 2 oligodendrogliomas generally demonstrate a more indolent trajectory, with survival frequently extending beyond ten to fifteen years, even in the setting of subtotal resection [1,3,4]. These distinctions remain clinically meaningful, guiding expectations for time-to-treatment escalation, informing radiographic surveillance intervals, and shaping long-term functional counseling in a disease where cumulative treatment burden and survivorship considerations are central [6].

The standard treatment paradigm for oligodendroglioma involves maximal surgical resection followed by adjuvant radiation and chemotherapy [6]. Although temozolomide is widely employed in clinical practice, the PCV regimen remains the preferred systemic therapy approach. PCV mediates its therapeutic effects through tumor cell cytotoxicity via DNA alkylation, reliant primarily on MGMT promoter hypomethylation [7]. Landmark trials, including EORTC 26951 and RTOG 9402, demonstrated that the addition of PCV to radiation therapy significantly improves survival in patients with 1p/19q-codeleted tumors. However, PCV is accompanied with substantial toxicity; grade 3 or higher myelosuppression is common, and neurotoxicity often necessitates treatment discontinuation [8].

In contrast, IDH inhibitors are targeted therapies that selectively act on mutant IDH enzymes that drive progression in oligodendrogliomas. These IDH mutations lead to accumulation of the oncometabolite 2-hydroxyglutarate (2-HG), which promotes tumor initiation and progressive growth, and they are the target of new therapy for oligodendroglioma and of IDH-mutant gliomas [9]. Vorasidenib, a dual IDH1/2 inhibitor, has demonstrated significant progression-free survival in the phase III INDIGO trial and, as of August 2024, holds FDA approval for patients aged 12 years and older with grade 2 astrocytoma or oligodendroglioma. Early-phase studies report that 22.8% of patients experience grade ≥ 3 adverse events, of which elevations in hepatic transaminases predominate, suggesting that the toxicity profile differs markedly from that of PCV therapy [10].

Despite advances in the literature, there remains gaps in our understanding of these new targeted systemic therapies. Notably, no direct comparative studies have been conducted between these two treatment options, limiting our ability to evaluate new options in comparison to current standard of care. This review intends to summarize current evidence for these treatment options, identify salient research gaps for upcoming work, and establish a framework to guide the design of future clinical trials.

## 2. Methods

This scoping review systematically mapped the existing literature on PCV chemotherapy and IDH inhibitors used in the treatment of IDH-mutant oligodendrogliomas. A scoping review was chosen to capture the extent of existing research and identify literature gaps, given heterogeneity in the data. Comprehensive searches were conducted across PubMed, Embase, Scopus, Web of Science, ClinicalTrials.gov, and the Cochrane Library. The search included all eligible studies published from database inception through 7 March 2025, utilizing search terms including “oligodendroglioma”, “PCV chemotherapy”, and “IDH inhibitors”, with no language or location restrictions applied.

**Figure 1:**
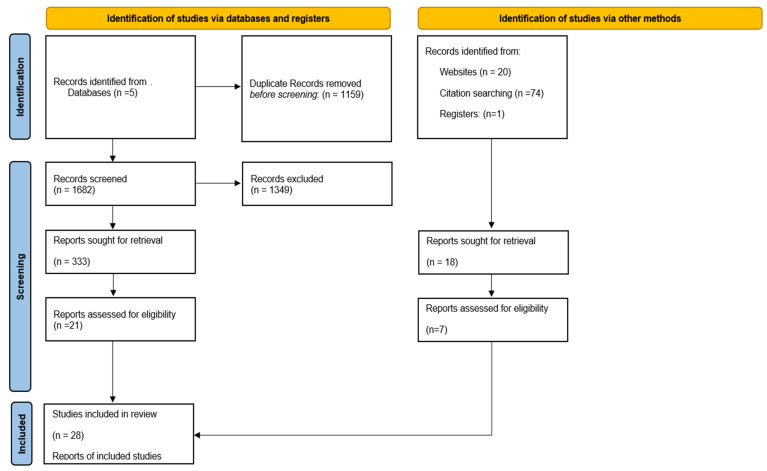
PRISMA flowsheet.

Eligible studies included clinical trials (all phases), observational studies, and case series (>5 patients) reporting adult patients (≥18 years) with oligodendroglioma diagnoses treated with PCV chemotherapy or IDH inhibitors. In studies that enrolled mixed glioma populations, we extracted only the data explicitly attributable to oligodendrogliomas. The final dataset therefore reflects 406 oligodendroglioma cases across all study designs. The IDH inhibitors included in this study were vorasidenib, ivosidenib, and AGI-5198. Eligible studies were required to report at least one clinical outcome of interest, including progression-free survival (PFS), overall survival (OS), grade ≥ 3 adverse events, or quality of life metrics. Exclusion criteria were preclinical studies, narrative reviews, editorials, opinion articles, and studies that did not report distinct data for oligodendrogliomas from other glioma subtypes. A PRISMA flowsheet is included in Figure 1, The study was registered in PROSPERO system with number CRD420251233894.

Two reviewers independently screened titles, abstracts, and full texts using Covidence software. Initial screening agreement was 92.3% (κ = 0.84), with conflicts resolved through discussion and consensus. Data extraction included study design, sample size, IDH mutation subtype, 1p/19q codeletion status, treatment details, median follow-up, progression-free survival, overall survival, objective response rates, adverse events (grade ≥ 3), quality of life measures, and resistance mechanisms. Studies were categorized by treatment modality and study design to enable a descriptive analysis of efficacy, safety, and resistance patterns. The preceding elements of this study design were conducted in accordance with PRISMA-ScR guidelines. We qualitatively evaluated key limitations including sample size, design heterogeneity, and risk of bias in existing work.

## 3. Results

### 3.1. Study Selection

A total of 1682 records were identified in the initial database search, from which 1349 were excluded for duplicate entries or failing the initial title screening. The remaining 333 citations were sought for retrieval, with 21 publications meeting all the criteria for review. In addition, 95 records were identified via other methods, including websites (*n* = 20), citation searching (*n* = 74), and registers (*n* = 1). Of the 95, 18 reports were sought for retrieval, and 7 reports were eligible. Consequently, 28 studies were included in the review.

### 3.2. Treatment Efficacy Profiles

**Figure 2:**
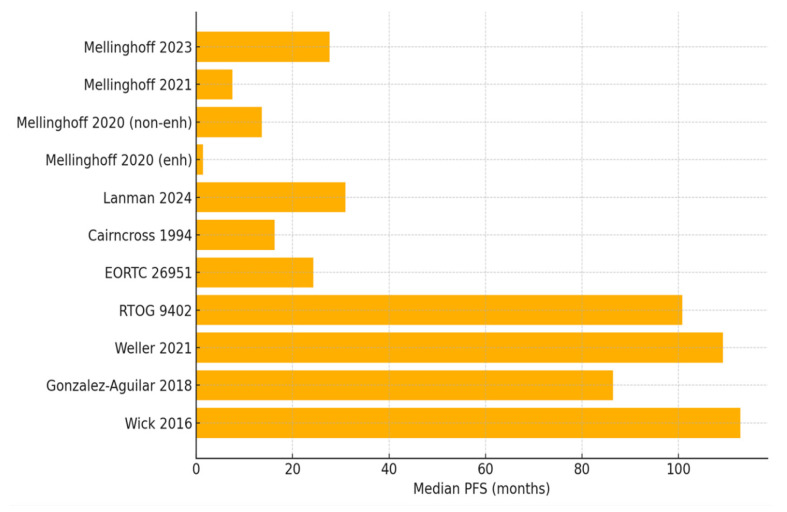
Median progression-free survival (PFS) across studies comparing IDH inhibitors and PCV-based therapies in IDH-mutant oligodendrogliomas [10,11,12,13,14,15,16,17,18,19,20].

**Figure 3:**
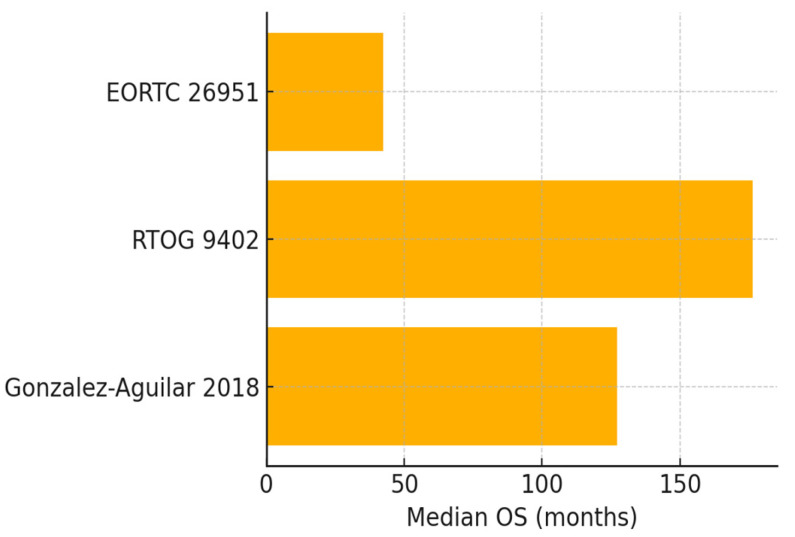
Median overall survival (OS) in studies of PCV-based therapy for IDH-mutant oligodendrogliomas [15,16,19].

Table 1 shows evidence regarding the efficacy of these therapies, with outcome measures depicted further in Figure 2 (PFS) and Figure 3 (OS). Below, the main high-quality data in the literature are highlighted and discussed.

#### 3.2.1. Evidence Profile of IDH Inhibitors

Evidence on IDH inhibitors is primarily derived from early-phase clinical trials conducted between 2020 and 2024. In the phase III INDIGO trial, vorasidenib significantly extended PFS with a median of 27.7 months (95% CI: 17.0–not reached) versus 11.1 months (95% CI: 10.2–13.8) in placebo controls (HR = 0.39, 95% CI: 0.27–0.56; *p* < 0.001). A subgroup analysis in the oligodendroglioma cohort demonstrated progression in 21.6% of patients receiving vorasidenib, as opposed to 47.6% of patients receiving the placebo treatment with 14.2 median follow-up [10]. In a retrospective analysis of ivosidenib, there was a median PFS of 31 months (95% CI: 19.2–42.8), with non-enhancing tumors showing longer PFS [13]. In phase I trials of vorasidenib, disease control rates reached 90.9% in non-enhancing tumors compared to 56.7% in enhancing tumors [11], with a number needed to treat (NNT) for a PFS benefit of 3.2 (95% CI: 2.4–4.8). Overall survival data remain limited, with the median follow-up in INDIGO being 14.2 months. When juxtaposed with the PFS range observed in PCV cohorts (24.3 months to 8.4 years), vorasidenib achieves comparable short-term disease control, though without the mature long-term survival evidence currently available for PCV.

#### 3.2.2. Evidence Profile of PCV Chemotherapy

PCV chemotherapy data are derived from trials conducted primarily in the 1990s–2010s. RTOG 9402 demonstrated a median OS of 14.7 years (95% CI: 10.9–18.2) for PCV plus RT versus 7.3 years (95% CI: 6.2–9.2) for RT alone in codeleted tumors (HR 0.59, 95% CI: 0.42–0.83; *p* = 0.003) [16]. EORTC 26951 showed a median OS of 42.3 months (95% CI: 33.1–56.8) versus 30.6 months (95% CI: 24.4–43.2) with PCV addition (HR 0.75, 95% CI: 0.60–0.94; *p* = 0.012). The NNT for OS benefit was 5.1 (95% CI: 3.8–8.2). The PFS was also improved in EORTC 26951 with 24.3 months in the PCV group versus 13.2 months without PCV (HR 0.66, 95% CI: 0.52–0.83; *p* < 0.001) [15]. These trials have long-term follow-up data exceeding 10 years. Across both trials, median PFS ranged from 24.3 months to 8.4 years (depending on grade and codeletion status), providing a benchmark for disease control duration. These mature PFS data allow for a more direct comparison for early PFS outcomes in IDH inhibitor studies.

### 3.3. Current Treatment Guidelines

The 2025 ASCO-SNO Rapid Recommendation Update addresses the role that vorasidenib has played following its FDA approval in August 2024. The current guideline provides conditional recommendations (1.2.1 and 1.5.1) that vorasidenib may be offered to patients with grade 2 IDH-mutant oligodendrogliomas where, after one or more surgeries, further treatment with radiation and chemotherapy has been or can be deferred [21]. This recommendation is based on high-quality evidence from the INDIGO trial demonstrating improved PFS (HR 0.39, 95% CI: 0.27–0.56) and prolonged time to next intervention (HR 0.26, 95% CI: 0.15–0.43) [10]. For grade 3 oligodendrogliomas, guidelines maintain for treatment with PCV plus radiation as the standard care based on long-term survival data, with no recommendation for vorasidenib in this population [21].

Importantly, the guidelines highlight several key considerations regarding the INDIGO trial’s strict inclusion criteria versus the broader FDA approval. The trial required measurable non-enhancing disease (≥1 cm^2^) and excluded patients with nodular enhancement, while the FDA approval allows patients with gross total resection [10,21]. The trial also mandated a 1–5-year window post-surgery, which the approval does not restrict [10]. Our analysis of contemporary cohorts suggests that only 30–40% of newly diagnosed grade 2 oligodendrogliomas would meet the original INDIGO criteria. The guidelines note insufficient data to recommend vorasidenib for patients who have received prior radiation or chemotherapy, those with measurable enhancement, or grade 3–4 disease. In terms of complication avoidance, regular hepatic monitoring is required every 2 weeks for the first 2 months and then monthly thereafter. The guidelines note that PCV toxicity can impact patient selection, and they recommend careful assessment of performance status and organ function [21]. MGMT methylation testing is recommended for treatment planning, although its role in decision-making regarding the potential use of an IDH inhibitor remains undefined.

### 3.4. Treatment Toxicity Profiles

Table 2 shows evidence regarding toxicity related to these therapies. Below, the main high-quality data are highlighted and discussed.

#### 3.4.1. Toxicity Profile of IDH Inhibitors

IDH inhibitors demonstrated grade ≥ 3 adverse events in 22.8% of patients in INDIGO, which were primarily severe ALT (9.6%) or AST elevations (8.9%). Treatment discontinuation due to adverse events occurred in 1–3% of patients. The NNH for grade ≥ 3 adverse events was 10.9 (95% CI: 6.8–24.3) [10]. Other IDH inhibitors including olutasidenib [22] and DS-1001 [23] had a 42% rate of grade ≥ 3 AEs. Most adverse events were manageable with dose modifications.

#### 3.4.2. Toxicity Profile of PCV Chemotherapy

In RTOG 9402, the pre-radiation PCV regimen was associated with frequent acute toxicities, most often myelosuppression, mood or cognitive changes, and peripheral or autonomic neuropathy. Two early deaths (1.37%) occurred from PCV-induced neutropenia. Just over half of patients (54%) were able to complete all four treatment cycles, and 20% discontinued therapy due to toxicity. The study did not specify the rate of vincristine-related neuropathy, provide a number-needed-to-harm estimate, or detail the frequency of the 75% dose modification threshold. No cases of therapy-related leukemia or severe cognitive decline were reported on long-term follow-up [8]. A combined final analysis of EORTC 26951 and RTOG 9402, with 18 to 19 years of follow-up, reaffirmed that PCV in addition to radiotherapy improves progression-free and overall survival, particularly in 1p/19q-codeleted tumors. These long-term data highlight both the regimen’s durable benefit and the need for continued monitoring of delayed treatment effects [8,16].

### 3.5. Quality of Life and Functional Outcomes

The absence of standardized patient-reported outcome (PRO) data represents a critical gap in existing evidence that impacts our ability to provide optimal patient-centered care. Among 28 included studies, only 3 (10.7%) collected any PRO data, and none used validated instruments consistently. The available data suggest different impacts on daily functioning between treatments, though robust comparisons are not possible. For IDH inhibitors, limited PRO data from INDIGO showed stable or improved quality of life scores in 78% of patients at 6 months [10]. For PCV, retrospective analyses indicate that 45–60% of patients report persistent fatigue, cognitive difficulties, or functional limitations during treatment [8]. The lack of systematic PRO collection using tools like EORTC QLQ-C30 and QLQ-BN20 has negative implications on informed decision-making. Future trials must mandate comprehensive PRO assessment given the extended survival in oligodendroglioma and the profound differences in treatment burden.

### 3.6. Resistance Mechanisms and Clinical Implementation

Table 3 shows evidence regarding mechanisms of therapy resistance in IDH inhibitors and PCV therapy. Below, the main data and themes are highlighted.

#### 3.6.1. IDH Inhibitors

Resistance mechanisms for IDH inhibitors remain under investigation. Preclinical data suggest that secondary IDH mutations and NOTCH1 alterations may confer resistance [29]. Cell-cycle gene co-mutations correlated with shorter PFS in exploratory analyses. Currently, no validated biomarker panels exist for patient selection beyond IDH mutation confirmation. MGMT-unmethylated tumors may theoretically benefit from earlier IDH inhibitor use given poor PCV response, though this requires prospective validation [7]. ctDNA monitoring approaches are under development at some academic centers, but there is not one standard approach, and this is not performed as part of standard-of-care clinical approaches [30].

#### 3.6.2. PCV Chemotherapy

The 1p/19q codeletion is the primary predictive biomarker for PCV chemotherapy response in gliomas, with codeleted tumors (oligodendrogliomas) showing dramatically improved survival compared to other non-codeleted gliomas [31,32]. MGMT promoter methylation serves as a prognostic marker associated with longer patient survival in gliomas but lacks predictive value for response to PCV specifically [33,34]. CDKN2A deletion has been associated with poor PCV response in some studies, although this requires further study and validation [31,35]. Factors leading to early progression within 12 months and treatment resistance mechanisms are not well understood, with ongoing research investigating molecular predictors of treatment response and recurrence in oligodendrogliomas.

### 3.7. Limitations of Current Evidence

**Figure 4:**
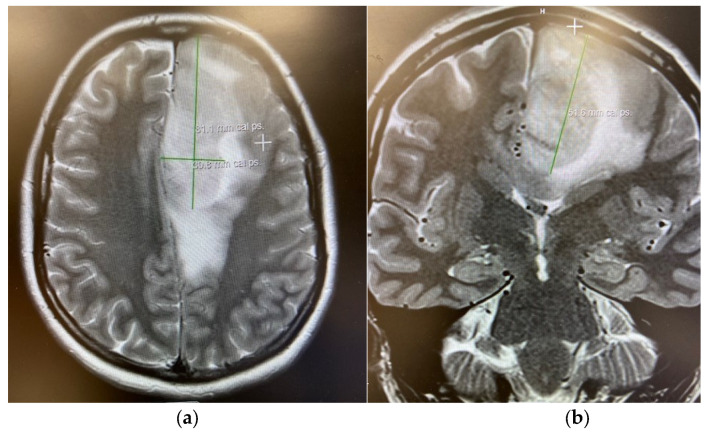
MRI images from clinical case. MRI brain demonstrates T2 hyperintense lesion and surrounding infiltration on an axial (**a**) and coronal (**b**) image on this axial T2 sequence.

**Figure 5:**
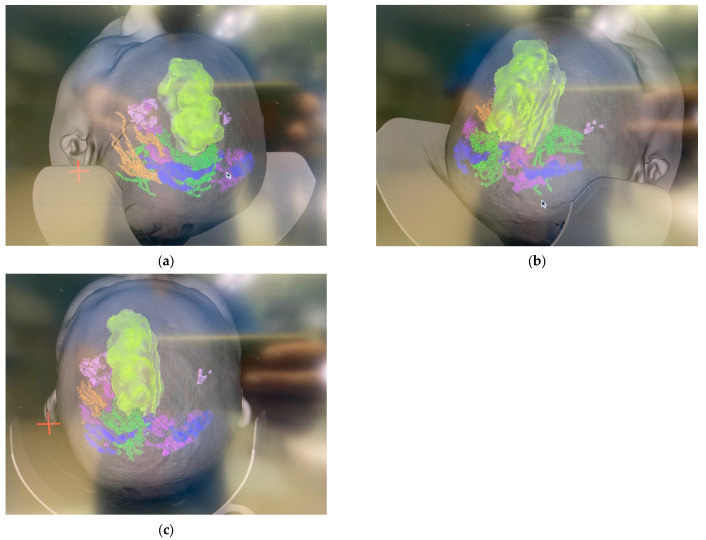
(**a**–**c**) Intraoperative neuronavigation-assisted connectomics. Colored mapped images depict left-sided large frontal mass with displacement of the fiber tracts/fasciculus and the motor tracts.

Table 4 presents evidence gaps with existing IDH inhibitor and PCV therapy studies. Several fundamental limitations preclude us being able to make definitive conclusions about optimal treatment selection. Most critically, the absence of any head-to-head comparative trials makes relative efficacy assessment impossible. The temporal gap between PCV trials (1990s–2010s) and IDH inhibitor trials (2020s) introduces confounding from evolved diagnostics, imaging, and supportive care. Different primary endpoints (OS for PCV, PFS for IDH inhibitors) further complicate interpretation. IDH inhibitor trials have insufficient follow-up to assess long-term survival, with median follow-up under 2 years versus >10 years for PCV trials. Patient populations differ substantially in molecular characterization completeness and prior treatment exposure. The near-complete absence of PRO data represents a fundamental failure in patient-centered trial design. These limitations necessitate extreme caution in clinical application and highlight the urgent need for properly designed comparative trials. To contextualize the therapeutic data presented above, we include representative MRI and intraoperative images (Figure 4 and Figure 5) that depict the characteristic radiographic appearance of oligodendrogliomas and their relationship to adjacent eloquent white-matter tracts [36].

## 4. Discussion

This review characterizes the discrete evidence frameworks surrounding PCV chemotherapy and IDH inhibitors in the context of treating oligodendrogliomas, IDH-mutant 1p/19q codeleted gliomas. Given the substantial methodological divergence across available studies, direct comparisons between treatment modalities are difficult [38]. Accordingly, therapeutic profiles are examined and presented independently, with the intention of outlining the available data and elucidating knowledge gaps to guide future prospective studies.

### 4.1. Efficacy Contextualization

PCV chemotherapy remains the only treatment regimen supported by mature overall survival data from randomized trials, with median OS exceeding 14 years in 1p/19q-codeleted glioma populations (oligodendrogliomas). These data, derived from studies conducted over the past three decades, have firmly established PCV’s role in contemporary treatment algorithms. The 2025 ASCO-SNO guideline update continues to affirm this position, issuing a strong recommendation for PCV therapy in combination with radiation for patients with WHO grade 2 oligodendrogliomas (Recommendation 1.1), while permitting deferral in select cases with favorable prognostic features (Recommendation 1.2).

In contrast, IDH inhibitors have demonstrated promising early-phase activity with a median PFS of 27.7 months in the INDIGO trial. However, it remains unclear whether these PFS gains will translate into overall survival benefits on the level seen with the PCV regimen. The median follow-up time of 14.2 months in the INDIGO trial is, unfortunately, an insufficient timeframe from which to draw conclusions about long-term outcomes in these patients. Preliminary data suggest that non-enhancing tumors respond more favorably to IDH inhibition. Of importance, the strict eligibility criteria employed in INDIGO may constrain generalizability, as only an estimated 30–40% of patients in typical clinical practice are estimated to meet these same criteria [10].

A further consequence of this evidentiary asymmetry is the absence of mature overall survival benchmarks for IDH inhibitors, a limitation that fundamentally restricts their interpretability within the therapeutic landscape of oligodendroglioma. Although these agents demonstrate compelling early disease control activity, the INDIGO trial’s median follow-up of 14.2 months provides only a narrow window into a disease whose natural history frequently extends beyond a decade. Short-interval progression-free survival, while valuable as an early indicator of biologic activity, cannot substitute for long-term survival metrics in a tumor type defined by protracted clinical trajectories and delayed treatment effects. This lack of durable outcome data carries immediate implications for clinical practice. Current guidelines position vorasidenib primarily in contexts where radiation and chemotherapy have been intentionally deferred, reflecting not a demonstrated equivalence to PCV but rather the incompleteness of the survival evidence available to date. The constraint is even more pronounced in grade 3 oligodendrogliomas, where the therapeutic threshold for altering standard-of-care regimens is higher and where broad adoption of IDH inhibitors remains premature without extended survival outcomes showing durability that approaches that of established cytotoxic therapy.

The absence of long-term survival data therefore represents more than a methodological deficiency; it is a structural barrier to full integration of IDH inhibition across the spectrum of oligodendroglioma care. Addressing this gap will require future trials to incorporate prolonged follow-up intervals capable of capturing not only overall survival but also time to malignant transformation, late treatment-related morbidity, and the durability of tumor control across successive treatment epochs. Harmonized eligibility criteria, uniform endpoints, and robust longitudinal surveillance will be essential for determining whether early radiographic benefit translates into the long-term clinical outcomes necessary to redefine therapeutic sequencing for these patients.

### 4.2. Impact of Study Design Heterogeneity on Interpretation

The comparative interpretation of outcomes associated with PCV chemotherapy and IDH inhibition is defined by the inherent heterogeneity of the available evidence. The randomized phase III trials evaluating PCV, conducted from the 1990s through the early 2010s, enrolled diagnostically diverse populations, many of which predated contemporary molecular classification standards. By contrast, the evidence supporting IDH inhibitors is derived primarily from modern early-phase trials that preferentially include molecularly well-characterized, non-enhancing tumors in patients who have received limited or no prior therapy. This divergence produces structural imbalances in baseline risk, patterns of prior treatment, imaging requirements, and the selection of primary endpoints. PCV trials rely on overall survival as the central outcome and benefit from more than a decade of follow-up, whereas IDH inhibitor studies emphasize short-term progression-free survival without comparable long-term surveillance. These methodological differences limit the validity of indirect comparisons and may artificially magnify or obscure perceived differences in efficacy or toxicity. Rigorous, prospective, head-to-head trials that employ harmonized eligibility criteria and standardized outcomes will be necessary to determine the true comparative effectiveness of these treatment strategies.

### 4.3. Toxicity and Patient-Reported Outcome Gaps

There are significant differences in treatment tolerability between the two regimens; however, the absence of standardized patient-reported outcomes impairs physician–patient decision-making. PCV chemotherapy is associated with grade ≥ 3 adverse events in 65–70% of patients, often resulting in early discontinuation primarily due to persistent neuropathy [8]. These tolerability issues raise concern that effectiveness in clinical practice may fall short of trial-based efficacy. In contrast, IDH inhibitors show grade ≥ 3 adverse events in 22.8% of patients. This is arguably a more favorable safety profile consisting of reversible transient elevations in hepatic transaminases, resulting in less frequent treatment cessation.

The paucity of PRO data in the current literature represents a critical deficiency for the current evidence base. Among the studies included in this review, only 10.7% of included studies reported any PRO data, and none employed standardized or systematic collection methods. This limitation is particularly important for oligodendroglioma as a generally low-grade glioma with prolonged patient survival compared to glioblastoma, so quality of life through the post-treatment period is an important outcome. We recommend that future trials prioritize the inclusion of validated PRO scales by including EORTC QLQ-C30 and QLQ-BN20 collection at baseline and throughout treatment and follow-up. Without such outcome data, cost–utility analyses incorporating quality-adjusted life years (QALYs) remain infeasible, and this limits the ability to perform comprehensive evaluation of therapeutic value in a patient-centered context.

The near absence of patient-reported outcome data across oligodendroglioma studies represents a major deficiency in the contemporary literature. Among the twenty-eight studies included in this review, only three collected any PRO measures, and none employed validated instruments in a consistent, longitudinal manner. This gap is particularly consequential in a tumor type characterized by prolonged survival, where quality of life, neurocognitive function, fatigue, and treatment-related morbidity exert significant influence on long-term well-being. These considerations are central when comparing a toxic regimen such as PCV with a better tolerated targeted therapy, since radiographic metrics alone cannot capture the experiential burden of treatment. Standardized PRO instruments, including the EORTC QLQ-C30 and QLQ-BN20, will be essential for future trials to meaningfully evaluate patient-centered outcomes. Without such measures, any attempt at cost–utility analysis or comparative effectiveness assessment remains fundamentally limited. A more deliberate integration of PRO methodology is therefore necessary to align future research with the priorities of modern neuro-oncology.

### 4.4. Biomarker-Driven Selection

Current biomarker implementation differs between treatments. For PCV, MGMT methylation testing is validated, widely available (USD 500–700), and cost-effective for predicting response [39]. For IDH inhibitors, reliable biomarkers to predict response or resistance have not been identified. IDH mutation status alone determines eligibility, but no validated markers guide treatment decisions. Although IDH inhibitors have shown clinical efficacy in various cancers, their high cost (approximately USD 33,693 per 28-day cycle for ivosidenib) relative to traditional chemotherapy highlights the importance of identifying predictive biomarkers to optimize patient selection and treatment outcomes [40].

### 4.5. Our Clinical and Scientific Recommendations Based on Existing Data

#### 4.5.1. Clinical Decision Framework

We propose a framework that best utilizes the available data while acknowledging its limitations. We emphasize that this framework requires prospective validation and will evolve with emerging data.

Newly diagnosed oligodendrogliomas:

- For MGMT methylated tumors in patients with a good performance status: PCV plus radiation remains strongly recommended per the 2025 ASCO-SNO guidelines given the proven OS benefit.
- For patients where radiation/chemotherapy has been deferred and not indicated postoperatively: Vorasidenib may be offered (conditional recommendation 1.2.1) based on INDIGO trial.
- For MGMT unmethylated tumors or patients with poor PCV tolerance: Consider IDH inhibitor, acknowledging uncertain long-term benefits.
- For non-enhancing disease meeting the INDIGO criteria: Discuss both options, emphasizing PCV’s proven OS versus IDH inhibitor’s tolerability.
- For patients with enhancement or grade 3 disease: No vorasidenib recommendation; PCV plus radiation remains standard.

Recurrent oligodendrogliomas:

- For progression post-PCV: IDH inhibitor reasonable given different mechanism of action.
- For progression post-IDH inhibitor: Limited data; consider PCV if previously untreated.

#### 4.5.2. Agenda for Future Research

Below, we outline and prioritize recommendations for future studies to address the gaps in existing data that we have outlined and discussed, including specific study designs and projected timelines:

Critical Priority (address within 2–3 years)

1. Head-to-head randomized trial comparing IDH inhibitors versus PCV (phase III, *n* = 500, primary endpoint OS, projected completion 2032).
2. Standardized PRO collection using EORTC QLQ-C30/BN20 in all trials (implementation feasible immediately).
3. Optimal sequencing trial: upfront IDH inhibitor→PCV versus PCV→IDH inhibitor (phase II, *n* = 200, projected completion 2030).

High Priority (address within 3–5 years)

1. Biomarker-driven patient selection beyond MGMT status (multi-institutional discovery cohort, *n* = 1000, validation *n* = 500).
2. Long-term neurocognitive outcomes (longitudinal cohort with annual testing, *n* = 300, 10-year follow-up).
3. Cost-effectiveness modeling incorporating quality-adjusted life year analyses (decision analysis study).

Medium Priority (address within 5–7 years)

1. Combination strategies (phase I/II dose-finding, multiple arms).
2. Enhancement status validation as predictive biomarker (imaging-stratified trial, *n* = 150).
3. Resistance mechanism characterization from clinical samples (translational study embedded in trials).

In summary, the evidence gaps identified mandate a coordinated research agenda. Most urgently, a head-to-head randomized trial comparing PCV and IDH inhibitors with OS as the primary endpoint is essential. This trial must include comprehensive PRO assessment, biomarker stratification, and pharmacoeconomic analysis. Sample size calculations suggest *n* = 500 for 80% power to detect HR = 0.75, requiring international collaboration.

Standardized PRO implementation can begin immediately without awaiting new trials. We recommend mandating EORTC QLQ-C30/BN20 in all oligodendroglioma studies, with collection at baseline, every 3 months during treatment, and long-term follow-up. Electronic PRO platforms can minimize burden while maximizing data quality.

Biomarker development requires systematic tissue collection, both tumor and blood, in all trials with centralized analysis. It will be valuable to evaluate MGMT promoter methylation, cell-cycle alterations, and existing ctDNA alterations in plasma. This tissue resource will be fundamental for identifying novel biomarkers in future multiomic integrated analyses. In tissue, this may have utility for improved patient prognostication and for the development of new targeted therapies. In plasma, new biomarkers may allow for non-invasive tumor diagnosis and/or serial monitoring for progression.

## 5. Conclusions

This scoping review delineates the evolving evidence landscape surrounding PCV chemotherapy and IDH inhibitors in oligodendrogliomas while underscoring that direct cross-trial comparisons remain difficult due to profound methodological heterogeneity across three decades of research. PCV chemotherapy has the only long-term survival data, with median overall survival surpassing 14 years in appropriately selected populations. However, its substantial toxicity profile continues to limit treatment duration. Conversely, IDH inhibitors have demonstrated promising early-phase efficacy, achieving a median progression-free survival of 27.7 months with markedly fewer severe adverse events. Whether these short-term benefits will ultimately match or exceed the established survival outcomes of PCV remains uncertain.

The current evidence base is constrained by the absence of head-to-head comparative trials, lack of standardized patient-reported outcomes, and insufficient biomarker-driven guidance in management decision, all of which limit our ability to provide evidence-based therapeutic decision-making. Until these gaps are addressed, clinical choices must be grounded in transparent discussion of both the proven yet toxic advantages of PCV and the more tolerable, albeit unproven, promise of IDH inhibition. We provide our recommendations on navigating this decision-making process. Future research should prioritize comparative effectiveness studies integrating patient-reported outcomes, long-term follow-up, and biomarker stratification. Only through such rigor can the field advance toward genuinely personalized, patient-centered care that balances survival benefit with quality of life in this uniquely enduring disease.

## Figures and Tables

**Table 1:** Therapy efficacy data.

Study	Treatment	Population (Gliomas)	Median PFS	Median OS	ORR	DCR	Study Type	Notes
Mellinghoff et al., 2023 [10]	Vorasidenib vs. Placebo	*n* = 331 (Oligo = 172)	27.7 vs. 11.1 mo	NR	10.7% vs. 2.5%	94.0% vs. 91.4%	Phase III RCT (ongoing)	Included both oligodendroglioma and astrocytoma patients; efficacy consistent across histologies.
Mellinghoff et al., 2021 [11]	Vorasidenib	*n* = 52 (Oligo = 16) (22 non-enh; 30 enh)	36.8 mo (non-enh); 3.6 mo (enh)	NR	ORR: 18.2% (non-enh); 0% (enh)	non-enh: 90.9%; enh: 56.7%	Phase I	Non-enhancing tumors had better outcomes.
Mellinghoff et al., 2020 [12]	Ivosidenib	*n* = 66 total (Oligo ≥ 3) (35 non-enh; 31 enh)	13.6 mo (non-enh); 1.4 mo (enh)	NR	2.9% (non-enh) 0% (enh)	88.6% (non-enh) 45.2% (enh)	Phase I	Non-enhancing tumors had better outcomes.
Lanman et al., 2024 [13]	Ivosidenib	*n* = 74 total (Oligo = 39)	31 mo	NR	**7.7%**	82%	Retrospective	Non-enhancing better outcomes.
Cairncross et al., 1994 [14]	PCV	*n* = 24 oligo	16.3 mo	NR	75%	92%	Prospective phase II	High initial response rate.
EORTC 26951 [15]	RT vs. RT + PCV	Phase III; *n* = 368 anaplastic oligodendroglial tumors (1p/19q-codeleted = 80)	13.2 vs. 24.3	30.6 vs. 42.3	NR	NR	Phase III RCT	Long-term survival benefit.
RTOG 9402 [16]	RT vs. RT + PCV	*n* = 291 AO/AOA (known codeleted: 59)	2.9 vs. 8.4 y (specifically codeleted)	7.3 y vs. 14.7 y	NR	NR	Phase III RCT	Codeleted subgroup favored PCV.
Weller et al., 2021 [17]	PCV, TMZ, Surgery, or Wait and Scan	*n* = 142 Oligos (PCV *n* = 30)	PCV: 9.1 y	NR	NR	~100% (PCV)	Retrospective cohort	PCV group showed the longest PFS.
Kacimi et al., 2025 [18]	RT + PCV vs. RT + TMZ	*n* = 305 Oligo	10 yr PFS: 67% (PCV) vs. 35% (TMZ)	10 yr OS: 72% (PCV) vs. 60% (TMZ)	NR	NR	Prospective observational	PCV/RT significantly improved OS and PFS.
Gonzalez-Aguilar et al., 2018 [19]	RT + PCV vs. RT + TMZ	*n* = 48 Oligo	PCV: 7.2 y; TMZ: 6.1 y	PCV: 10.6 y; TMZ: 9.2 y	PCV: 80.9%, TMZ: 70.3%	NR	Retrospective	PCV/RT significantly improved OS and PFS.
Wick et al., 2016 (NOA-04) [20]	Initial RT vs. initial PCV vs. initial TMZ	*n* = 274 anaplastic gliomas (Oligo = 68)	RT: 8.67 y; PCV: 9.4 y; TMZ: 4.46 y	RT: n/r (9.95–n/r); PCV: n/r (8.19–n/r) y; TMZ: 8.09 y	NR	NR	Phase III RCT	In IDH-mut/1p19q-codel tumors, PCV showed longer PFS than TMZ.

Abbreviations: AO = anaplastic oligodendroglioma; AOA = anaplastic oligoastrocytoma; CCNU = lomustine; CI = confidence interval; CR = complete response; DCR = disease control rate; HR = hazard ratio; IDH = isocitrate dehydrogenase; MGMT = O6-methylguanine-DNA methyltransferase; mo = months; NNT = number needed to treat; NNH = number needed to harm; NR = not reached; ORR = objective response rate; OS = overall survival; PC = procarbazine plus lomustine; PCV = procarbazine, lomustine, and vincristine; PFS = progression-free survival; RT = radiotherapy; TMZ = temozolomide.

**Table 2:** Therapy-induced toxicity data.

Study	Treatment	Population Gliomas	Grade ≥ 3 AE Rate	Common AEs	Discontinuation Rate	Evidence Type	Notes
Mellinghoff et al., 2023 [10]	Vorasidenib vs. Placebo	Oligo *n* = 172	22.8% vs. 13.5%	LT ↑, AST ↑, GGT ↑	31.5% (Vora) vs. 57.1% (Placebo)	Phase III RCT	Mostly liver enzyme elevations
Mellinghoff et al., 2020 [12]	Ivosidenib	*n* = 66 total (Oligo ≥ 3) (35 non-enh; 31 enh)	19.7% overall	Headache, Nausea, Fatigue, Vomiting, etc.	77.3%	Phase I	Most stopped due to progression
De La Fuente et al., 2022 [22]	Olutasidenib	*n* = 26 (Oligo = 6)	42%	Nausea, Fatigue, Diarrhea, Vomiting, etc.	77%	Phase Ib/II Prospective clinical trial	50% dropped out of study due to death
Natsume et al., 2022 [23]	DS-100	*n* = 47 (Oligo = 15)	42.6%	Skin hyperpigmentation, diarrhea, pruritus, etc.	83%	Phase I dose-escalation	Many discontinued due to progression
Lanman et al., 2024 [13]	Ivosidenib	*n* = 74 (Oligo = 39)	8%	Elevated CK, QTc prolongation, diarrhea, transaminitis	1%	Retrospective	Well tolerated
EORTC 26951 [15]	RT vs. RT + PCV	Phase III; *n* = 368 anaplastic oligodendroglial tumors (1p/19q-codeleted = 80)	Greater than or equal to 32%	Neutropenia, Thrombocytopenia, anemia, nausea, etc.	52% PCV discontinued	Phase III RCT	Phase III RCT
RTOG 9402 [16]	RT vs. RT + PCV	*n* = 291 AO/AOA (known codeleted: 59)	65%	Acute myelosuppression (neutropenia, thrombocytopenia), cognitive or mood change, peripheral/autonomic neuropathy, intractable vomiting, hepatic dysfunction, and severe allergic rash	70% PCV discontinued	Phase III RCT	Phase III RCT
Rincon-Torroella et al., 2024 [24]	RT + PCV vs. RT + TMZ vs. RT	*n* = 277 oligodendroglioma	NR	Peripheral neuropathy, thrombocytopenia leukopenia, rash	0% fully discontinued (PCV)	Retrospective cohort	Many modifications but no full discontinuations in PCV group. ~75% required PCV dose adjustment
Tabouret et al., 2015 [25]	PCV	*n* = 89 (Oligo = 64)	46%	Anemia Thrombocytopenia Neutropenia	61.8%	Retrospective	Toxicity-driven discontinuation negatively impacted survival (HR = 5.09)
Gonzalez-Aguilar et al., 2018 [19]	RT + PCV vs. RT + TMZ	*n* = 48 Oligo	PCV: 42.8% vs. TMZ: 11.1%	Leukopenia thrombocytopenia, etc.	PCV: 57.2%, TMZ: 19.8%	Retrospective	PCV group had higher hematologic toxicity, leading to lower completion rate
Ahn et al., 2022 [26]	PC vs. PCV	*n* = 59, Oligo = 9)	PCV: ≥70.4% vs. PC: ≥20%	Anemia, thrombocytopenia, neutropenia, peripheral neuropathy (only in PCV group), etc.	PCV: 68.2% vs. PC: 26.7%	Single-institution retrospective	Lower toxicity with PC vs. PCV, but also potential efficacy differences

Abbreviations: AE = adverse event; ALT = alanine aminotransferase; AST = aspartate aminotransferase; BN20 = Brain Cancer Module of the EORTC QLQ; CK = creatine kinase; CTCAE = Common Terminology Criteria for Adverse Events; DCR = disease control rate; Enh = contrast enhancing tumor; IDH = isocitrate dehydrogenase; mo = months; ORR = objective response rate; PC = procarbazine and lomustine; PCV = procarbazine, lomustine, and vincristine; PFS = progression-free survival; PRO = patient-reported outcome; QTc = corrected QT interval; RT = radiotherapy; TMZ = temozolomide; AO = anaplastic oligodendroglioma; AOA = anaplastic oligoastrocytoma; CCNU = lomustine; CI = confidence interval; CR = complete response; DCR = disease control rate; HR = hazard ratio; IDH = isocitrate dehydrogenase; MGMT = O6-methylguanine-DNA methyltransferase; mo = months; NNT = number needed to treat; NNH = number needed to harm; Non-enh = non-enhancing tumor; NR = not reached; ORR = objective response rate; OS = overall survival; RT = radiotherapy.

**Table 3:** Data on therapy resistance.

Study	Treatment	Population	Resistance Factor	Evidence Type	Notes
Mellinghoff et al., 2021 [11]	Vorasidenib	Phase I; *n* = 52 total (Oligo = 16)	Possible isoform switching or incomplete 2-HG suppression	Early-phase clinical data	PFS differed by tumor enhancement status
Mellinghoff et al., 2020 [12]	Ivosidenib	Phase I; *n* = 66 total (Oligo ≥ 3)	Co-mutations in cell-cycle genes shorten PFS in non-enhancing gliomas	Exploratory biomarker analysis	Non-enhancing tumors fared better
Natsume et al., 2022 [23]	DS-1001	Phase I; *n* = 47 gliomas (15 Oligo)	Not clearly identified secondary mutation; drug retained 2-HG suppression	Phase I trial w/correlative data	Partial data on resistance
Spitzer et al., 2024 [27]	Ivosidenib/Vorasidenib	Preclinical and translational (*n* = 7 + in vivo) (3 oligos)	NOTCH1 mutation dampened differentiation response	Preclinical/translational	Astrocytic differentiation rescue overcame partial inhibitor resistance
EORTC 26951 [15]	RT vs. RT + PCV	Phase III; *n* = 368 anaplastic oligodendroglial tumors (1p/19q-codeleted = 80)	Oligodendrogliomas showed little OS/PFS gain→relative resistance to PCV	Phase III RCT	Benefit restricted to codeleted subset; PCV toxicity limited full 6-cycle completion
RTOG 9402 [16]	RT vs. RT + PCV	*n* = 291 AO/AOA (known codeleted: 59)	1p/19q-**intact** oligodendrogliomas derived no OS benefit from PCV→RT (OS 2.6 y vs. 2.7 y)	Phase III RCT	Confirms codeletion as predictive; intact tumors relatively resistant
NOA-04 (Wick et al., 2016) [20]	PCV vs. TMZ vs. RT	*n* = 274 anaplastic gliomas (Oligo = 68)	Unmethylated MGMT promoter in IDH-wild-type/CIMP-negative tumors→limited benefit from alkylating chemotherapy	Phase III RCT w/subgroups	Better prognosis in IDH-mut/codel; resistance in IDH-wt/CIMP-neg
Van den Bent et al., 1998 [28]	PCV (Standard vs. Intensif.)	Recurrent Oligo/OA post-radiation (*n* = 52)	Early relapse (<1 yr after initial surgery ± RT)→low CR/PR rate (~25%) to PCV	Retrospective multicenter	Suggests chemo-resistance in early relapse

Abbreviations: AO = anaplastic oligodendroglioma; CIMP = CpG island methylator phenotype; CR = complete response; IDH = isocitrate dehydrogenase; MGMT = O6-methylguanine-DNA methyltransferase; NNT = number needed to treat; OA = oligoastrocytoma; OS = overall survival; PCV = procarbazine, lomustine, and vincristine; PFS = progression-free survival; PR = partial response; RT = radiotherapy; TMZ = temozolomide.

**Table 4:** Limitations of key therapy studies.

Study	Treatment	Identified Gap/Limitation
All IDH Inhibitor Trials	Vorasidenib, Ivosidenib, etc.	No direct PCV comparison; long-term OS data lacking; limited resistance data
RTOG 9402/EORTC 26951 [8]	RT + PCV	No IDH inhibitors studied; biomarker data limited; focused only on RT + PCV
Bell et al., 2020 [37]	RT ± PCV	Focused on high-risk LGG; post hoc IDH/codel analysis; side effect data limited
Lanman et al., 2024 [13]	Ivosidenib (retrospective)	Retrospective; possible selection bias; no OS data; inconsistent assessments
Tabouret et al., 2015 [25]	PCV (retrospective)	Multicenter; no comparison to TMZ/IDH inhibitors; retrospective toxicity reporting
Natsume et al., 2022 [23]	DS-1001 (phase I)	No biomarker analysis for resistance; small sample; early-phase design
NOA-04 (Wick et al., 2016) [20]	RT→chemo vs. chemo→RT (PCV/TMZ)	Randomization not specific to Oligo; subgroup lacked power
All PCV vs. TMZ Comparisons	PCV ± RT vs. TMZ ± RT	Retrospective; no RCTs in pure 1p/19q-codel, IDH-mut Oligos; toxicity underreported
Spitzer et al., 2024 [27]	Preclinical IDH inhibitors	Small tumor line sample

Abbreviations: AE = adverse event; IDH = isocitrate dehydrogenase; MGMT = O6-methylguanine-DNA methyltransferase; NR = not reported; OS = overall survival; PCV = procarbazine, lomustine, and vincristine; PFS = progression-free survival; PRO = patient-reported outcome; RCT = randomized controlled trial; RT = radiotherapy; TMZ = temozolomide.

## Data Availability

All data extracted and analyzed in this review were obtained from publicly available published studies. No new data were created, and no unpublished datasets were generated or analyzed. All sources are cited within the manuscript.
